# The effect of cardiac resynchronization therapy on functional capacity based on cardiopulmonary exercise testing: a systematic review and meta-analysis

**DOI:** 10.1093/ehjopen/oeaf176

**Published:** 2025-12-29

**Authors:** Jhiamluka Solano, Nithusa Rahunathan, Dominic L Sykes, Gedoni Eni, Leyan Edhem, Klaus K Witte

**Affiliations:** Northern Lincolnshire and Goole NHS Trust, Scunthorpe General Hospital, Scunthorpe DN15 7BH, UK; Hull University Teaching Hospitals NHS Trust, Anlaby Rd, Hull HU3 2JZ, UK; Hull University Teaching Hospitals NHS Trust, Anlaby Rd, Hull HU3 2JZ, UK; Northern Lincolnshire and Goole NHS Trust, Scunthorpe General Hospital, Scunthorpe DN15 7BH, UK; Northern Lincolnshire and Goole NHS Trust, Scunthorpe General Hospital, Scunthorpe DN15 7BH, UK; Leeds Institute of Cardiovascular and Metabolic Medicine, University of Leeds, Leeds LS2 9JT, UK

**Keywords:** Cardiac resynchronization therapy, Cardiopulmonary exercise testing, Peak oxygen uptake, Ventilatory efficiency, Anaerobic threshold, Heart failure

## Abstract

**Aims:**

Cardiac resynchronization therapy (CRT) has a class 1a indication for patients with heart failure due to reduced ejection fraction (HFrEF) who also have conduction delay. Post-CRT management pathways are uncommon. Cardiopulmonary exercise testing (CPET) provides objective functional assessments and may serve as a valuable tool in assessing CRT response and guide device optimization. This systematic review and meta-analysis aimed to assess the effect of CRT on key CPET parameters and identify patients who may benefit from further intervention.

**Methods and results:**

A systematic search of MEDLINE, EMBASE, and Cochrane Central (May 2024) identified randomized controlled trials, non-randomized trials, and cohort studies evaluating changes in CPET post-CRT. Primary outcome was peak VO₂, with anaerobic threshold and ventilatory efficiency as secondary outcomes. Results were reported as standardized mean differences (SMD) and effect sizes using Cohen’s *d*.

**Results:**

Fourteen studies (12 cohort studies and 2 RCTs) involving 858 patients were included. CRT was associated with significant improvements in peak VO₂ (SMD = 0.62, 95% CI 0.19–1.05, *P* < 0.001), anaerobic threshold (SMD = 0.70, 95% CI 0.03–1.36, *P* = 0.04), and ventilatory efficiency (SMD = −0.45, 95% CI −0.68 to −0.21, *P* < 0.001). Considerable heterogeneity was noted, likely reflecting differences in exercise protocols, patient characteristics, and device programming.

**Conclusion:**

CRT improves exercise capacity and ventilatory efficiency, reinforcing its physiological benefits beyond cardiac remodelling. CPET may support personalized post-CRT care, including optimization of device programming, medications, and rehabilitation. Worsening CPET parameters may help identify patients progressing to advanced heart failure, allowing for timely care planning.

## Graphical Abstract

**Graphical Abstract:**
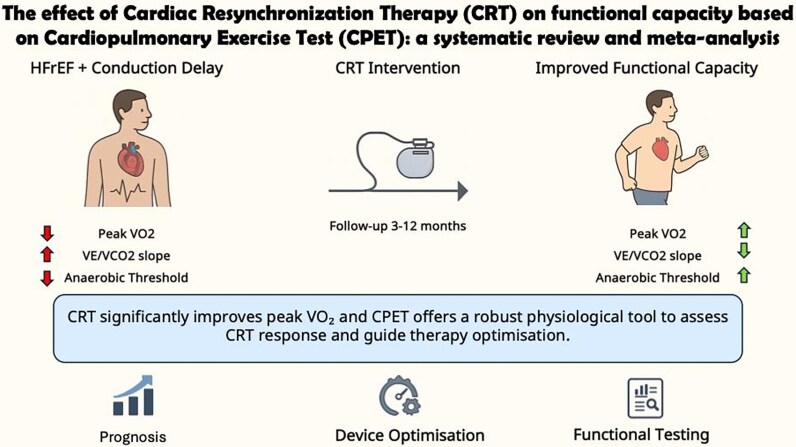

## Introduction

Heart failure (HF) represents a significant global health challenge, affecting around 64 million people worldwide and contributing to substantial morbidity, mortality, and healthcare expenditures.^1^ In Europe, the prevalence is 1–2% in adults and an incidence of about 3/1000 person-years (all age groups) or about 5/1000 person-years in adults.^2^ While advancements in pharmacological and device therapies have improved outcomes in HF,^3^ many patients continue to experience disabling symptoms and impaired functional capacity. Functional capacity can be assessed by cardiopulmonary exercise testing (CPET), providing peak and submaximal variables (peak oxygen uptake (pVO₂) and anaerobic threshold (AT)) and measures related to the underlying pathophysiology such as the slope relating ventilation to carbon dioxide output (VE/VCO₂).^4,5^

Cardiac resynchronization therapy (CRT) has a Class Ia recommendation for HF patients with reduced ejection fraction (HFrEF) and a QRS duration of >150 ms due to left bundle branch block (LBBB), and a Class IIa recommendation for individuals with a QRS duration of > 130 ms with LBBB and >150 ms with other conduction abnormalities.^2^ CRT leads to improved cardiac output and reverse left ventricular (LV) remodelling, improvements in symptoms and patient-oriented outcomes.^6,7^ However, the effect of CRT on functional capacity, particularly as assessed by CPET, is inconsistent and incompletely understood.^8,9^ Understanding this relationship is crucial, as changes in functional capacity correspond more closely with patient-orientated outcomes, including quality of life than LV remodelling.^11^

This systematic review and meta-analysis aim to describe the effect of CRT on functional capacity in patients with HF, using CPET-derived measures as outcomes, with the goal of providing data to help clinicians. Additionally, we explore the potential role of CPET in establishing and helping address variables that influence the disease modification effect of CRT.

## Methods

### Protocol registration

This systematic literature review and meta-analysis was performed in compliance with the ‘Preferred Reporting of Items for Systematic Reviews and Meta-analyses (PRISMA)’ guidelines and was prospectively registered on the ‘International Prospective Register of Systematic Reviews (PROSPERO)’ with the registration number CRD42024542614 (https://www.crd.york.ac.uk/PROSPERO/view/CRD42024542614).

### Eligibility criteria

The focus of the review was on CPET in people who had undergone CRT. Hence, we only included studies that published data on CPET before and after CRT. Furthermore, the following criteria had to be met for inclusion: (i) randomized controlled trials, non-randomized trials, or cohort studies and (ii) articles published in English. Studies without accessible abstract or full text, as well as studies that met one or more of the following criteria, were excluded (i) case reports, case series, systematic reviews, and meta-analyses and (ii) studies including patients with respiratory or skeletal muscle conditions as their primary diagnosis.

### Search strategy

The search was conducted in May 2024 using the MEDLINE, EMBASE, and Cochrane Central Register of Controlled Trials databases. The search terms used were (i) heart failure AND, (ii) cardiac resynchronization therapy OR CRT AND, (iii) cardiopulmonary exercise test OR exercise test OR CPET OR oxygen consumption AND, and (iv) functional capacity OR capacity OR exercise tolerance (see supplementary material online, *Figure S1*). There were no restrictions on status, year, or publications.

### Selection process

All identified publications were imported into Covidence, an online tool that streamlines parts of the systematic review process. Any duplicates were removed automatically by Covidence. Two independent reviewers initially selected studies based on their titles and/or abstracts, with discrepancies resolved by consensus. Any remaining duplicates were manually highlighted by the reviewers and removed. Two independent reviewers again screened full-text articles to find eligible studies. Both reviewers resolved conflicts between themselves by consensus.

### Outcomes of interest

Studies had to include at least one of the outcomes of interest to be included in the analysis. The primary outcome of interest was peak VO₂. Secondary outcomes of interest were anaerobic threshold and ventilatory efficiency.

### Data extraction

The data extraction process consisted of a standardized form designed in Microsoft Excel and piloted by both reviewers before use. Data recorded included first author, year of publication, study design (RCT or longitudinal cohort), duration of the study, number of participants (*n*), sex distribution, inclusion and exclusion criteria, mean age, effect of CRT on pVO₂, effect of CRT on ventilatory efficiency, effect of CRT on anaerobic threshold, reported *P*-values, and limitations as highlighted by the authors. Unreported data were displayed as no data (ND).

### Risk of bias assessment

The risk of bias was systematically assessed for all studies included in the meta-analysis, categorized into observational cohort studies and randomized controlled trials (RCTs). Two independent reviewers assessed the risk of bias in 12 of the studies using the ROBINS-I (Risk Of Bias In Non-randomized Studies—of Interventions) tool and in the remaining two using the Cochrane Risk-of-bias Tool on the Covidence website. All disagreements in the assessment were resolved by consensus.

For cohort studies, we evaluated bias across seven domains: confounding, participant selection, intervention classification, deviations from intended interventions, missing data, outcome measurement, and selection of reported results. In contrast, the assessment of RCTs focused on five key domains: randomization process, deviations from intended interventions, missing outcome data, measurement of outcomes, and selection of reported results.

### Data synthesis and calculation

We performed meta-analyses for all studies comparing the baseline pVO₂ mean and the follow-up pVO₂ mean, as well as the baseline and follow-up AT and ventilatory efficiency means. The analysis reports all effect sizes of each study as standardized mean differences (SMD) and presented as forest plots. The effect size was assessed using Cohen’s d, where values up to 0.2 indicate a small effect, between 0.2 and 0.8 indicate a moderate effect, and 0.8 or higher indicates a large effect. We describe skewed data as medians and interquartile ranges. All studies included are weighted by their sample size and variance, ensuring that larger, more precise studies contributed proportionally more to the pooled effect estimate. Heterogeneity across the included studies was evaluated using Cochran’s *Q* test, and the degree of inconsistency was expressed as the *I*² statistic. Meta-analyses were performed using IBM SPSS Statistics (version 28).

## Results

### Study selection

**Figure 1:**
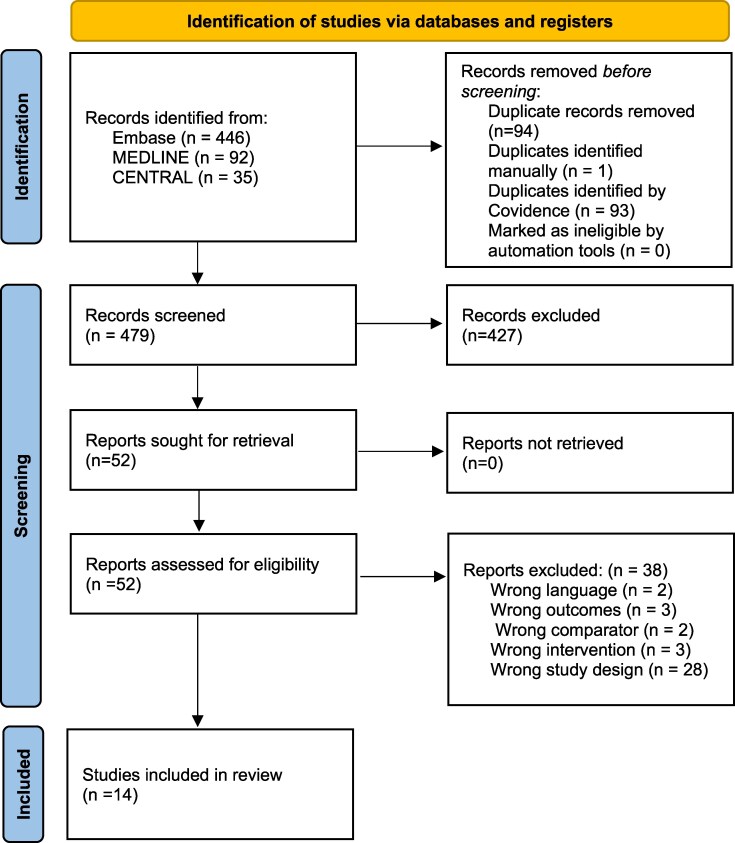
PRISMA flow diagram demonstrating study selection and exclusion.

The search from EMBASE, MEDLINE, and CENTRAL identified 573 studies, of which 94 were duplicates. A totatl of 479 studies were screened by their title and/or abstract, resulting in 51 studies for full-text review. Fourteen studies, comprising of 858 patients, were included in the final analysis of this review. The selection process is demonstrated in *Figure 1*, the PRISMA flow diagram. *Table 1* describes the study characteristics.

**Table 1:** Study characteristics^11–24^

Study	Study design	Number of participants	outcomes of interest	Follow up	Mean baseline pVO_2_ (mL/kg/min)
**Malfatto et al. 2005** ^12^	NCT pre and post clinical trial	31	pVO_2_, VE, AT	1–3 months and 12–15 months	10.5
**Celebi et al. 2012** ^11^	NCT pre and post clinical trial	136	pVO_2_, VE	9.4 months	14.7
**Jaussaud et al. 2012** ^13^	NCT pre and post clinical trial	10	pVO_2,_ VE	6 months	14
**Piepoli et al. 2008** ^14^	Randomized controlled trial	44	pVO_2_, VE	6 and 12 months	8.9
**Jaussaud et al. 2011** ^15^	NCT pre and post clinical trial	50	pVO_2_, VE, AT	6 months	13
**Tomczak et al. 2012** ^16^	NCT pre and post clinical trial	12	pVO_2_	6 months	12.9
**Maass et al. 2009** ^17^	NCT pre and post clinical trial	144	pVO_2_, AT	6 months	16.2
**Jaussaud et al. 2010** ^18^	NCT pre and post clinical trial	30	pVO_2_, VE	6 months	13
**Mastenrboek et al. 2016** ^19^	NCT pre and post clinical trial	84	pVO_2_, VE	6 months	15.5
**Chwyczko et al. 2008** ^20^	NCT pre and post clinical trial	27	pVO_2_, VE, AT	3–6 months	11.3
**Arora et al. 2012** ^21^	NCT pre and post clinical trial	76	pVO_2_, VE	6 and 12 months	11
**Auricchio et al. 2002** ^22^	NCT pre and post clinical trial	50	pVO_2_, VE, AT	3 months	1112^a^
**Wouters et al. 2022** ^23^	NCT pre and post clinical trial	31	pVO_2_, VE	3 months	16.4
**Wasserman et al. 2007** ^24^	Randomized controlled trial	192	pVO_2_, VE, AT	6 months	990^a^

pVO_2_, peak oxygen uptake; VE, ventilatory efficiency; AT, anaerobic threshold.

^a^mL/min.

### Effect of CRT on pVO₂

**Figure 2:**
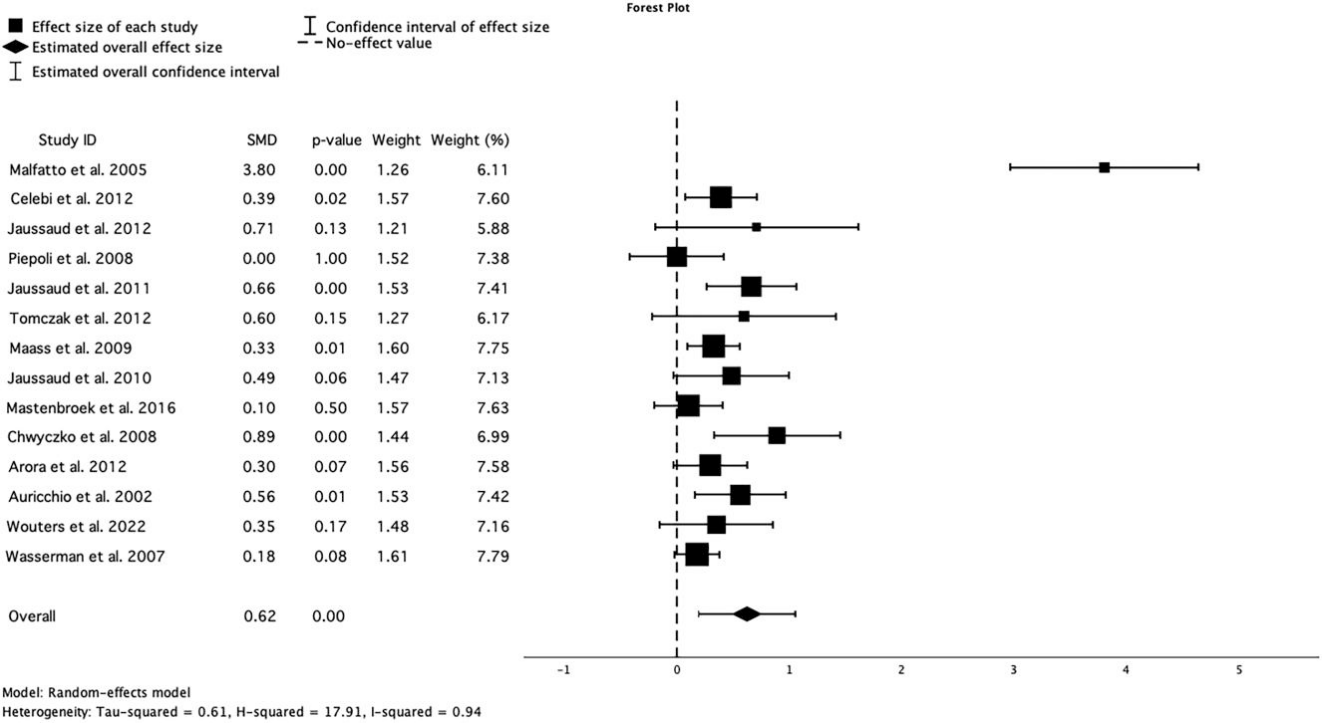
Forest plot for the meta-analysis of the effect of CRT on peak VO₂, 0 denotes null effect. SMD, standardized mean difference.

All 14 studies included baseline and follow-up (3–12 months after CRT) measures of pVO2. Meta-analysis demonstrated that a statistically significant improvement follows CRT in pVO₂ [SMD = 0.62 (CI 0.19–1.05; *P* < 0.001)]. Individual study results showed varying effect sizes, with some studies demonstrating substantial improvements and others reporting more modest changes. Malfatto *et al*.^12^ reported the highest effect size (SMD = 3.80, *P* < 0.001), while Piepoli *et al*.^14^ showed no significant effect (SMD = 0.00, *P* = 1.00) (*Figure 2*). Heterogeneity analysis revealed significant variability across studies (I² = 94%), suggesting substantial differences in patient populations, study designs, or intervention protocols.

### Effect of CRT on ventilatory efficiency

**Figure 3:**
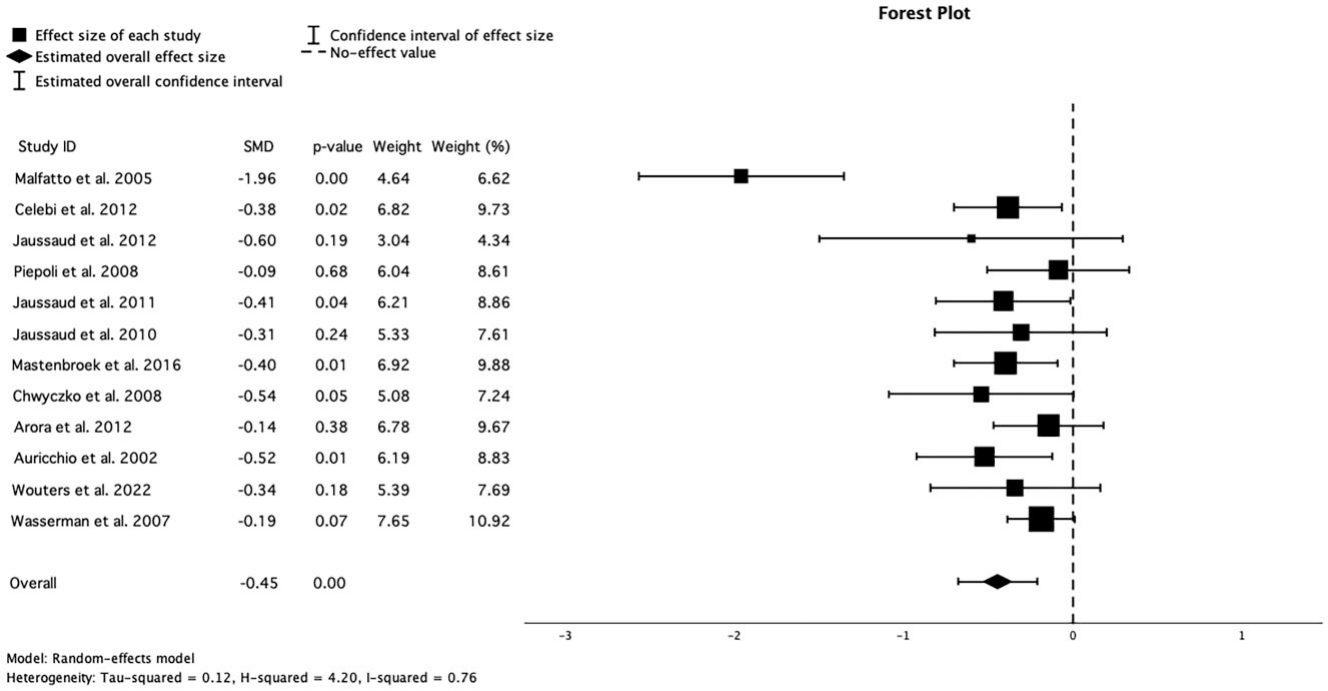
Forest plot for the meta-analysis of the effect of CRT on ventilatory efficiency, 0 denotes null effect. SMD, standardized mean difference.

Twelve studies included in this analysis measured ventilatory efficiency before and after CRT implantation. The pooled analysis showed a significant improvement in ventilatory efficiency (SMD = −0.45, CI −0.68 to −0.21, *P* < 0.001). The results across individual studies varied, with Malfatto *et al*.^12^ reporting the most substantial effect (SMD = −1.96, *P* < 0.001), while Piepoli *et al*.^14^ showed a minimal, non-significant change (SMD = −0.09, *P* = 0.68) (*Figure 3*). Heterogeneity analysis revealed significant variability among studies (*I*² = 76%), indicating some differences in study populations, methodologies, or intervention protocols.

### Effect of CRT on anaerobic threshold

**Figure 4:**
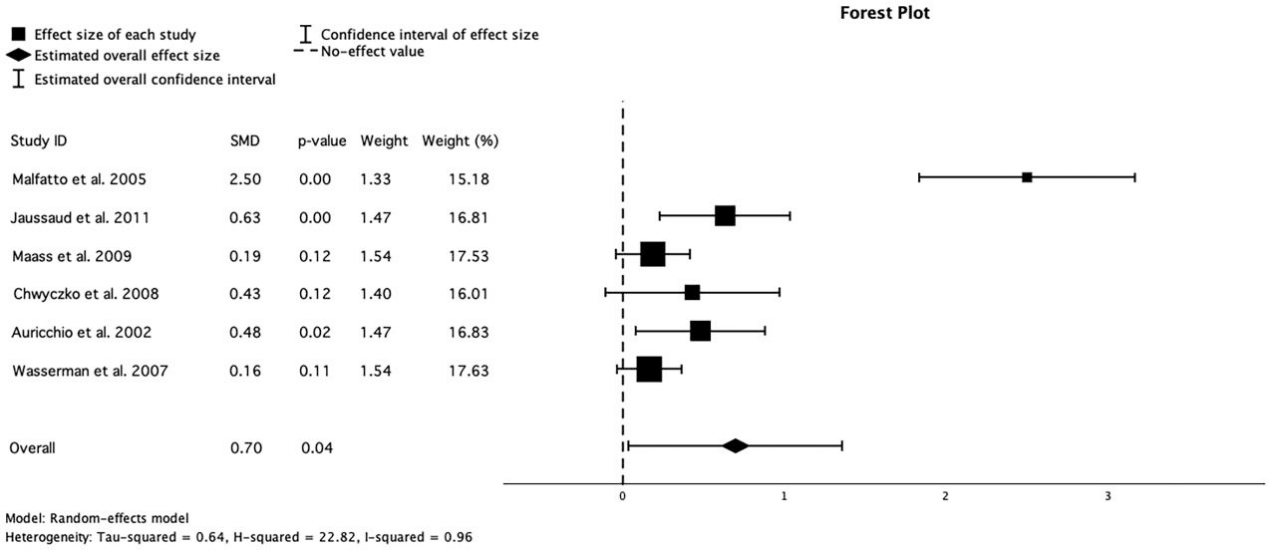
Forest plot for the meta-analysis of the effect of CRT on anaerobic threshold, 0 denotes null effect. SMD, standardized mean difference.

Six studies included in the analysis reported anaerobic threshold. There was a significant improvement in the anaerobic threshold demonstrated in the pooled analysis (SMD = 0.70, CI 0.03–1.36, *P* = 0.04). The individual study results varied, with Malfatto *et al*.^12^ showing the most pronounced effect (SMD = 2.50, *P* < 0.001), while Wasserman *et al*.^24^ reported a minimal, non-significant change (SMD = 0.16, *P* = 0.11) (*Figure 4*). Heterogeneity analysis revealed substantial variability among studies (*I*² = 96%), suggesting significant differences in study populations, methodologies, or intervention protocols.

### Risk of bias summary

Among the cohort studies (*n* = 12), the primary sources of bias were confounding and the classification of interventions. Nine studies exhibited significant bias due to confounding (see Supplementary material online, *Figure S1*), as these studies lacked rigorous methods to adjust for baseline differences between patient groups. Similarly, classification bias was evident in nine studies, potentially due to inconsistencies in defining the intervention groups or retrospective study designs. However, biases related to missing data, measurement of outcomes, and reporting of results were generally low across most cohort studies, suggesting a reliable reporting framework in these aspects.

In contrast, RCTs demonstrated a lower overall risk of bias, the studies shown achieved low-risk judgments across the domains. However, the randomization process for these studies was not routinely outlined in detail such that they were categorized as having ‘some concerns’ in this domain (see Supplementary material online, *Figure S2*). Although these trials adequately reported their randomization methods, the lack of detailed allocation concealment procedures raised potential risks of selection bias. Despite these concerns, the measurement of outcomes and reporting bias remained consistently low across all RCTs, indicating robustness in these aspects.

A funnel plot (see Supplementary material online, *Figure S3*) was generated to assess publication bias specifically for VO₂ peak, plotting standard error (SE) against standardized mean difference (SMD). For the VO₂ peak, the funnel plot demonstrated asymmetry, suggesting potential small-study effects. Egger’s test for publication bias yielded a statistically significant *P*-value (*P* = 0.014), indicating a high likelihood of publication bias. This suggests that smaller studies with negative or non-significant results may be underreported or missing from the analysis.

## Discussion

Cardiopulmonary exercise testing provides an objective, reproducible assessment of functional capacity, which is a key determinant of prognosis and quality of life in heart failure.^4,5^ Unlike imaging-based metrics such as left ventricular ejection fraction or echocardiographic remodelling parameters, which are themselves also related to prognosis,^25^ CPET directly evaluates oxygen kinetics, ventilatory efficiency, and cardiac output adaptation, reflecting whole-body functional improvements with CRT,^10,14^ providing additional and distinct outcome variables of importance to patients and relevant to their risk of future disease progression.^21^

Preprocedural CPET could provide information on the likely symptomatic benefit of CRT in an individual patient by identifying the major contributors to functional limitation.^4,5,10,14^ Peak VO₂, in general, correlates with survival in heart failure patients, while VE/VCO₂ slope is an independent marker of cardiovascular mortality and a measure of the pathophysiology underlying symptoms.^4,5^ Furthermore, the ability of CPET to capture dynamic physiological changes in response to CRT may help overcome the limitations of static echocardiographic and electrocardiographic markers, which are only modestly related to functional outcomes.^11^ More importantly, changes in CPET variables might identify additional targets to help improve patients’ status or at least optimize individual approaches to achieve disease stability.^10,14^

Peak oxygen uptake appears to rise significantly in many patients following CRT. Some studies^12,22^ reported marked improvements in pVO₂ within the first few months post-implantation, which were sustained during follow-up. Mastenbroek et al.^19^ identified baseline health status, non-ischaemic aetiology, and echocardiographic markers of favourable remodelling as predictors of a more robust functional response to CRT, in keeping with existing literature,^26,27^ which might not altogether be surprising given that in people with fewer comorbidities and less frailty, cardiac dysfunction is likely to contribute a greater proportion of their functional limitation. Conversely, patients with poorer initial exercise capacity often experience greater proportional increases in pVO₂, possibly because more advanced disease may provide a greater margin for functional recovery once coordinated ventricular contraction is restored.^21–24^ Discrepancies in follow-up duration and diversity of study design may also explain some variation in reported outcomes.

In addition to its effect on peak VO₂, CRT seems to enhance ventilatory efficiency, a variable that is less commonly reported but equally linked to long-term survival in heart failure patients as pVO2.^4,28^ Nevertheless, direct comparisons among studies are complicated by diverse measurement approaches, such as reporting slope vs. peak or nadir ratios. While Arora *et al*.^21^ found marked changes in ventilatory indices for CRT responders compared to non-responders, these improvements did not always correlate with baseline functional status, unlike the peak VO₂ patterns. Jaussaud *et al*.^15,18^ demonstrated that gains in ventilatory metrics can occur independently of significant left ventricular remodelling, indicating that the effect of CRT on respiratory control probably involves multiple mechanisms.^29,30^ Improved ventricular coordination may lessen sympathetic overactivity, decrease intrapulmonary shunting, improve skeletal muscle blood flow thereby optimizing the matching of ventilation to metabolic demands. Despite these encouraging results, differences in lung function at baseline, device settings and measurement protocols highlight the need for standardized methods in future studies.

Our analysis also reveals a trend towards elevated anaerobic threshold following CRT, indicating better submaximal exercise tolerance. While some studies document large relative improvements in AT,^12,15^ others find modest or non-significant changes,^17,22,24^ potentially reflecting heterogeneity in patient selection and CPET methodologies. Because AT is less influenced by patient motivation,^31,32^ it can serve as a more reliable indicator of physiologic adaptation than peak VO₂. However, the absence of uniform criteria for measuring AT complicated attempts to compare outcomes. Incorporating AT alongside peak VO₂ and ventilatory efficiency may offer a more comprehensive view of the physiological effects, as well as the impact on daily functioning, which often occurs at exercise levels below peak.

Although traditional markers such as NYHA class are widely used, they lack the precision needed to characterize functional capacity and treatment response fully. CPET provides greater physiological fidelity, enabling objective quantification of exercise limitation and cardiorespiratory reserve. Beyond baseline assessment, CPET could also guide post-CRT optimization strategies, including CRT programming adjustments (e.g. AV/VV delay modifications to improve synchrony),^18^ identifying adjunctive therapies (e.g. optimized pharmacological management, targeted exercise rehabilitation),^19^ and early identification of non-responders, prompting further investigations for AV delay, altered LV capture, anaemia, lung dysfunction, etc.^27,28^

A subset of patients may exhibit worsening CPET parameters post-CRT, raising concerns about non-response, disease progression, or suboptimal CRT programming (See *Table 2*).^12,17^ A decline in peak VO₂ or VE/VCO₂ slope or a failure to improve may indicate persistent or worsening dyssynchrony, possibly due to suboptimal lead positioning or inadequate CRT optimization.^22^ In some cases, this deterioration reflects the progression of heart failure, warranting consideration of advanced therapies such as LVAD or heart transplantation.^29^ Furthermore, a subset of patients, particularly those with suboptimal lead positioning, extensive myocardial scar, or atypical conduction patterns, may experience CRT-induced electromechanical inefficiencies, where pacing disrupts rather than enhances physiological contraction patterns.^23,24^

**Table 2:** Clinical implications of CPET deterioration after CRT

CPET deterioration pattern	Potential clinical interpretation	Suggested intervention
↓ **Peak VO₂ with stable VE/VCO₂**	Persistent left ventricular dysfunction, potential non-response to CRT	Consider optimizing pharmacologic therapy, re-evaluating CRT settings
↓ **Peak VO₂ and worsening VE/VCO₂**	Progression of heart failure, the potential need for advanced therapies	Evaluate for LVAD/transplant candidacy, intensify HF management
**Stable Peak VO₂ but worsening VE/VCO₂**	Increased ventilatory inefficiency, potential right ventricular dysfunction	Assess for pulmonary hypertension, consider right heart catheterization
↓ **Anaerobic threshold with unchanged peak VO₂**	Peripheral muscle deconditioning, suboptimal exercise tolerance	Encourage cardiac rehabilitation, assess for comorbidities
**Exercise oscillatory ventilation (EOV) appearance**	Severe HF prognosis, high risk of poor outcomes	Consider advanced HF therapies, reassess CRT efficacy

Close CPET monitoring could facilitate early intervention in these patients, potentially allowing for adjustments to CRT programming, medical therapy, or more aggressive intervention strategies.^31^ Understanding the specific patterns of CPET deterioration may provide insights into whether CRT adjustments, alternative pacing modalities, or device reprogramming are needed (*Table 2*).

This structured approach ensures that CPET is not only a predictive tool prior to CRT but also an ongoing monitoring strategy post-implantation, helping clinicians identify suboptimal responses early and tailor interventions accordingly. For patients who do not show functional improvement after CRT, further evaluation with advanced methods may help identify causes of non-response. Non-invasive and invasive CPET provide detailed functional and haemodynamic data, such as cardiac power output and pulmonary pressures, which, when combined with cardiac magnetic resonance imaging (CMR), can clarify disease mechanisms. Integrating CPET, CMR, echocardiographic, and electrocardiographic data may improve CRT response and guide personalized treatment adjustments.^33–35^

Moreover, there are other non-cardiac predictors for response to CRT, such as systemic inflammation, anaemia, and metabolic status. Elevated or high red cell distribution width (RDW), which reflects systemic inflammation, was independently associated with poorer functional and survival outcomes after CRT.^36^ As high RDW can result in impaired oxygen delivery, this could also be integrated with CPET parameters to explain the variability in improvements in pVO_2_ and ventilatory efficiency which could be used to assess response to CRT.

There are several limitations in this systematic review and meta-analysis. First, including both randomized and non-randomized observational cohort studies could introduce confounding factors. Second, substantial heterogeneity (*I*² > 75%) was observed across studies, likely due to variations in CRT programming, CPET protocols, and follow-up duration. Additionally, variability in CPET methodologies across studies—including differences between treadmill and cycle ergometry, which can yield up to 10–20% differences in peak VO₂—may have influenced the magnitude of observed effects.^36^ While we assessed for publication bias, the possibility of unpublished negative results remains a consideration. Due to the presence of publication bias, these findings should be interpreted with caution. Future research should address these gaps by minimizing selective publication and incorporating unpublished or negative findings to ensure a comprehensive synthesis of evidence.

## Conclusion

This systematic review and meta-analysis confirm that CRT significantly improves functional capacity in heart failure patients, as reflected by increases in peak VO₂, ventilatory efficiency, and anaerobic threshold. While the prognostic benefits of CRT extend beyond symptomatic improvement, cardiopulmonary exercise testing (CPET) may play a role post-CRT in distinguishing between cardiac and non-cardiac limitations and identifying factors contributing to a suboptimal response. Integrating CPET into a post-CRT care pathway could enhance patient management and therapy optimization.

## Supplementary Material

oeaf176_Supplementary_Data

## Data Availability

The data underlying this article will be shared on reasonable request to the corresponding author.
