# Supplementary material for: The effect of cardiac resynchronization therapy on functional capacity based on cardiopulmonary exercise testing: a systematic review and meta-analysis
Source: Eur Heart J Open. 2025 Dec 29;6(1):oeaf176. doi: 10.1093/ehjopen/oeaf176 (PMC12825617; doi:10.1093/ehjopen/oeaf176)
Supplement: oeaf176_Supplementary_Data [file oeaf176_supplementary_data.docx]

**Supplementary materials**

*
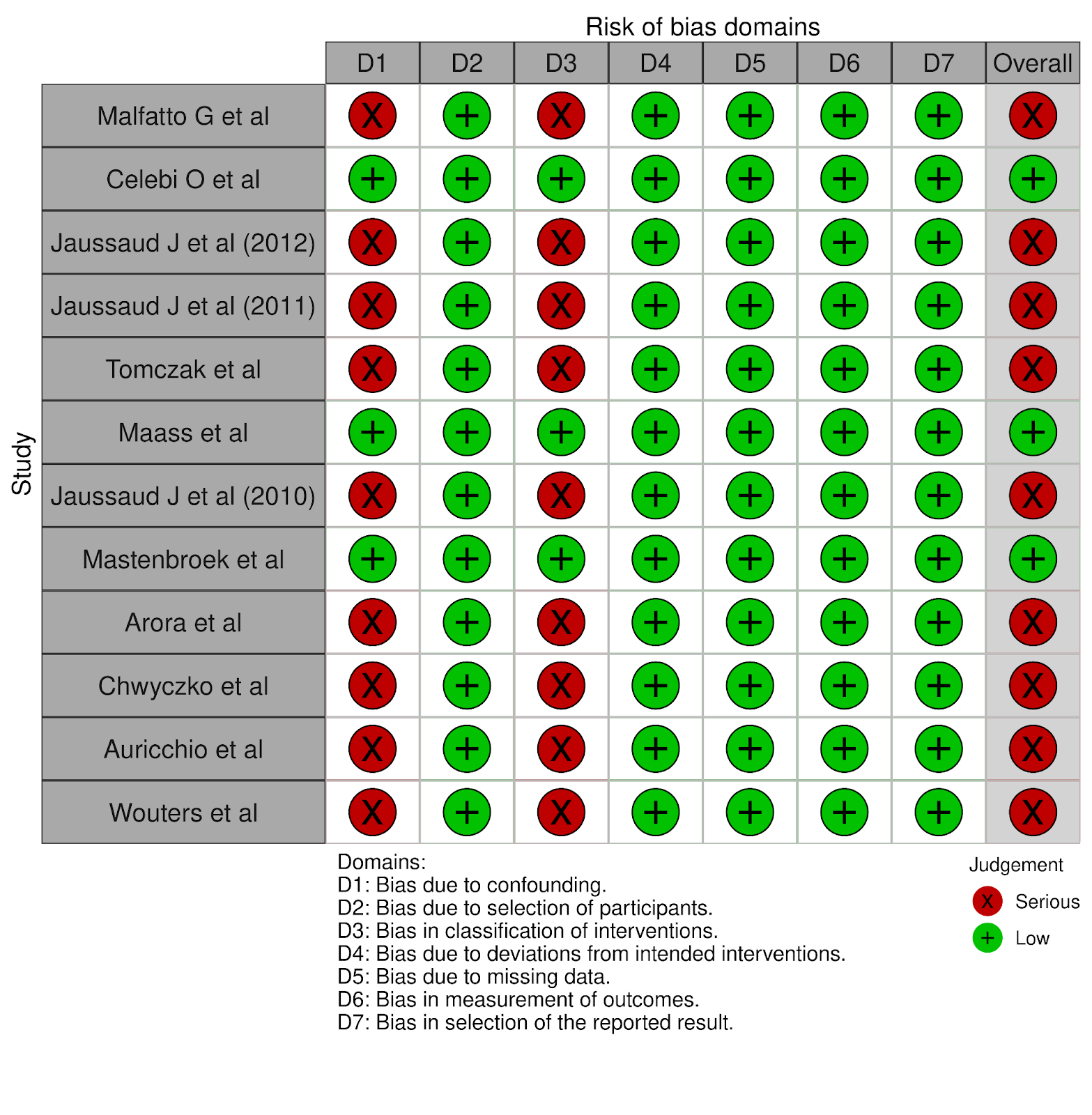
*

**Supplementary figure 1** *Cohort Studies Bias Assessment*

*
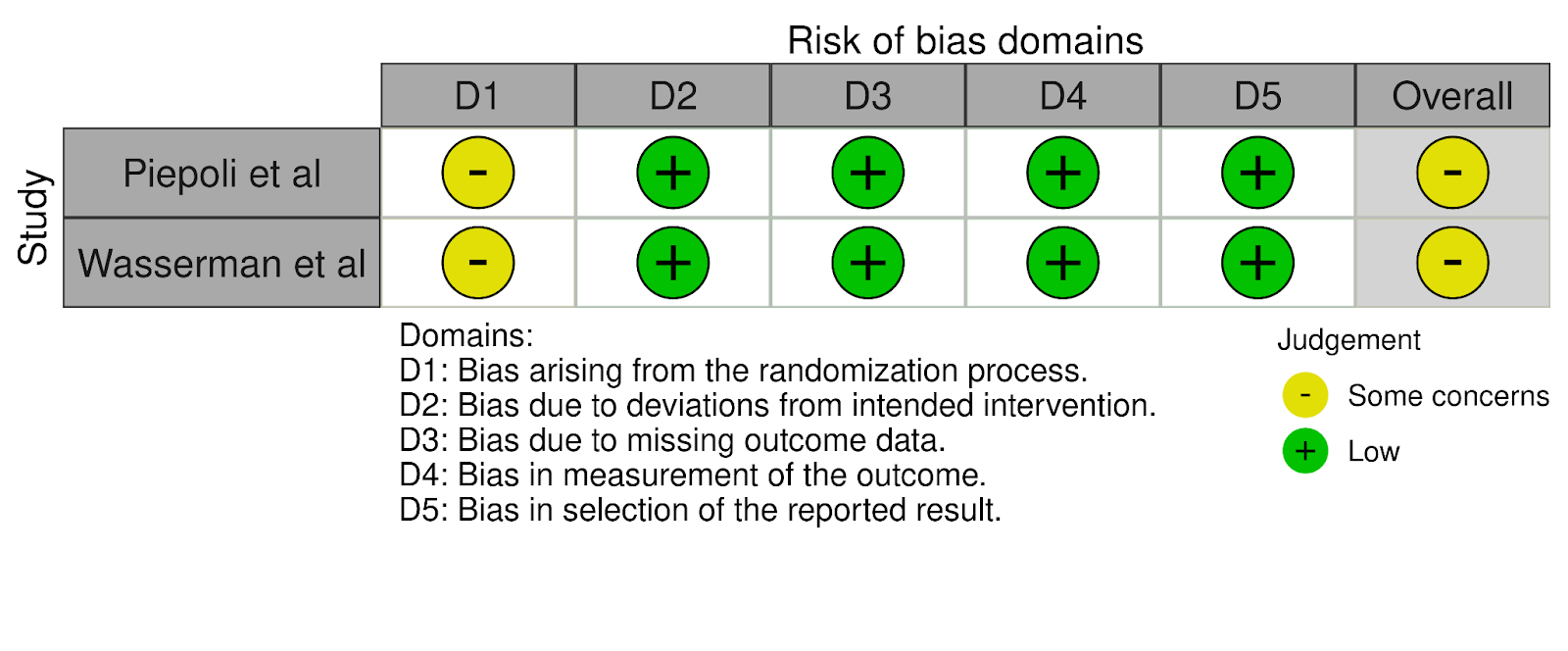
*

**Supplementary figure 2** *RCT Bias Assessment*


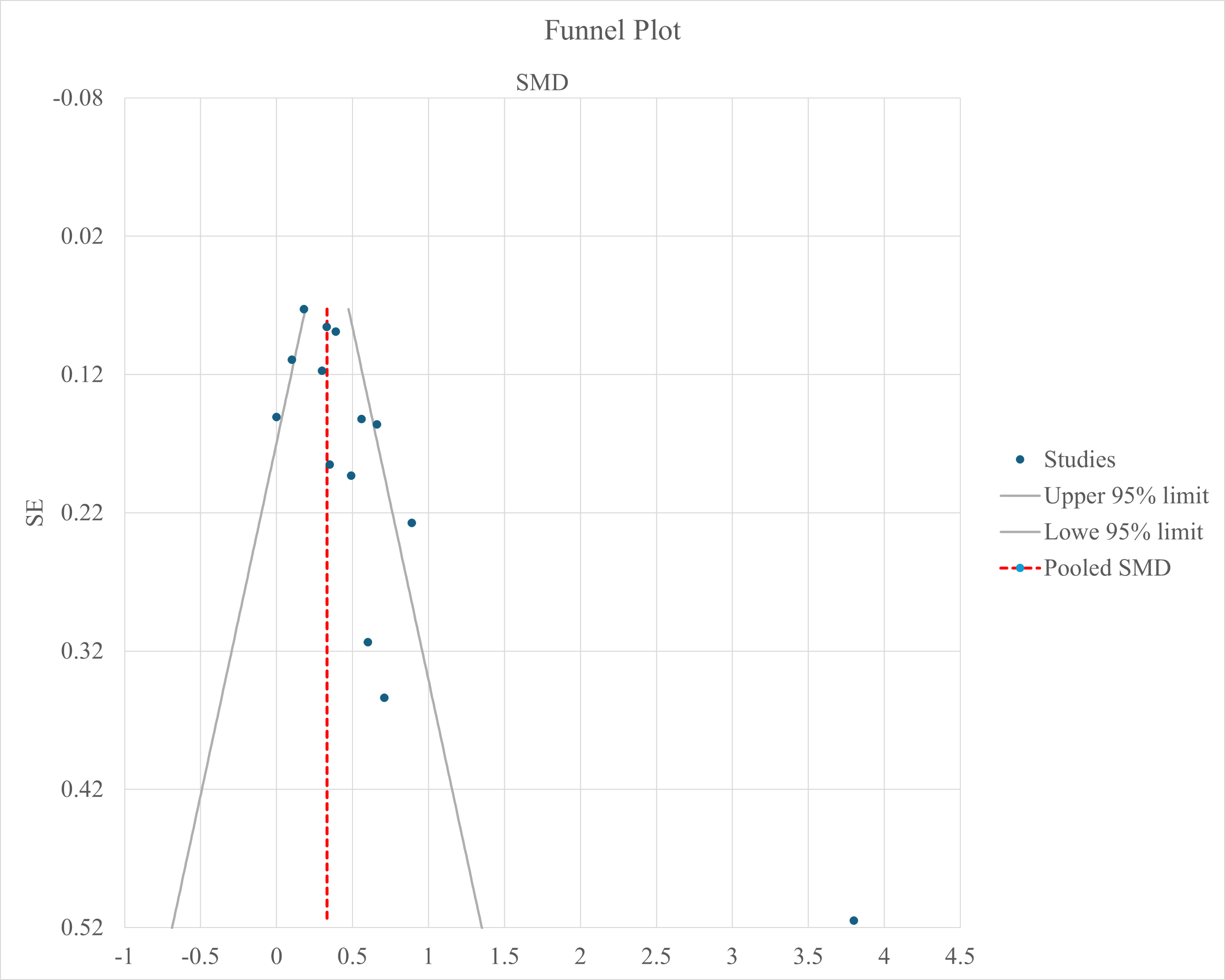


**Supplementary figure 3** *Funnel Plot assessing publication bias specifically for pVO₂ (Red line= Mean SMD [standardised mean difference])*
